# *NLRP7* Is Involved in the Differentiation of the Decidual Macrophages

**DOI:** 10.3390/ijms20235994

**Published:** 2019-11-28

**Authors:** Pei-Yin Tsai, Kuan-Ru Chen, Yueh-Chun Li, Pao-Lin Kuo

**Affiliations:** 1 Department of Obstetrics and Gynecology, National Cheng Kung University Hospital, College of Medicine, National Cheng Kung University, Tainan 70401, Taiwan; tsaipy@mail.ncku.edu.tw (P.-Y.T.); ggleon0119@gmail.com (K.-R.C.); 2 Laboratory of cytogenetic research, Lee Women’s Hospital, Taichung 40652, Taiwan

**Keywords:** *NLRP7*, macrophage, polarization, decidua

## Abstract

Macrophage polarization, regulated appropriately, may play important roles in successful pregnancy. In the face of the vital roles of decidua macrophages in pregnancy, it is insufficient to recognize the trigger of macrophage differentiation and polarization. We aimed to explore the link between the *NLRP7* gene and macrophage polarization in human deciduas. Here, we enrolled the endometrial tissues from eight pregnant women in the first trimester. We found that *NLRP7* was abundant in endometrial tissues and that *NLRP7* was expressed in decidual macrophages of the first-trimester pregnancy. *NLRP7* was predominately expressed in the decidual M2 macrophages, as compared with the M1 macrophages. Furthermore, our results suggest that *NLRP7* is associated with decidual macrophage differentiation. *NLRP7* over-expression suppresses the expression of M1 markers and enhances the expression of the M2 markers. Considering that *NLRP7* relates to decidualization and macrophage differentiation, we propose that *NLRP7* is a primate-specific multitasking gene to maintain endometrial hemostasis and reproductive success. This finding may pave the way for therapies of pathological pregnancies.

## 1. Introduction

The highly regulated dynamic immunologic process in pregnancy has been anticipated [1]. In the deciduas, the immune cells consist of natural killer (NK) cells (50–60%), macrophages (20–25%), T cells (10–20%), and dendritic cells (DCs) (1–2%) [2]. Several procedures of successful pregnancy, involving trophoblast invasion, tissue and vascular remodeling, and immune tolerance, are intricate by macrophages [2].

According to the plasticity and heterogeneity of macrophages, different functional macrophages are divided into M1 and M2 subtypes [3]. M1 macrophages are well known to play a major role in promoting inflammation. The classically activated macrophage with the capacity to present antigens to the adaptive immune system indicates the M1 subtype. M1-polarized macrophages are more effective at antigen clearance and switching T-cell responses toward T helper-1 immune response by a high expression of major histocompatibility complex class II, CD80, CD86, and IL-12 [4]. In addition, M2 populations, which are alternatively activated, have immunosuppressive capacities and are characterized by typical M2-associated markers, such as CD163, CD206, CD209, and IL-10. The M2 cells supply tissue remodeling and support Th2 or antibody-mediated immune responses [5].

The functions and phenotypes of macrophage with high plasticity are dependent on the microenvironment. Disruption of the uterine microenvironment can have profound effects on macrophage activity and can subsequently impact pregnancy outcomes. Many complications of pregnancy, including preeclampsia [6], fetal intrauterine growth restriction (IUGR) [7], spontaneous abortion [8], preterm labor [9], and intrauterine parasitic infections [10], could be associated with inappropriate macrophage polarization. M1 macrophages were abundant in the decidua of spontaneous abortions and unexplained recurrent spontaneous abortions, whereas the frequency of M2 macrophages was significantly higher in normal pregnancies [11]. The imbalance of M1/M2 macrophages has been considered one of the causes of pathological pregnancies. Appropriately regulated macrophage polarization has been considered an important factor of successful pregnancy [12]. 

Compared to the vital roles of decidua macrophages in pregnancy, little is known about the factors that trigger macrophage differentiation and polarization [13]. The NLR family have been shown to serve as innate immune sensors [14] and the NLR family member *NLRP7* (NACHT, leucine-rich repeat and PYD containing 7) has a well studied role in regulating immune responses [15,16,17]. In addition, *NLRP7* is up-regulated in the endometrial cells of pregnant women compared to non-pregnant women [18]. Up-regulated *NLRP7* had been found in both the nucleus and cytoplasm in the decidual stromal cells of human first-trimester endometrium [18]. In an in vitro decidualization model, *NLRP7* was up-regulated and only the short isoform translocated to the nucleus of endometrial stromal cells [18]. Given these facts, we were interested in exploring the link between the *NLRP7* gene and macrophage polarization in human deciduas. Investigations of decidual macrophages polarity and balance could help to clarify their roles in pregnancies and may pave the way to therapies of pathological pregnancies.

## 2. Results

### 2.1. NLRP7 Expressed in Decidual Macrophages of the First-Trimester Pregnancy

**Figure 1:**
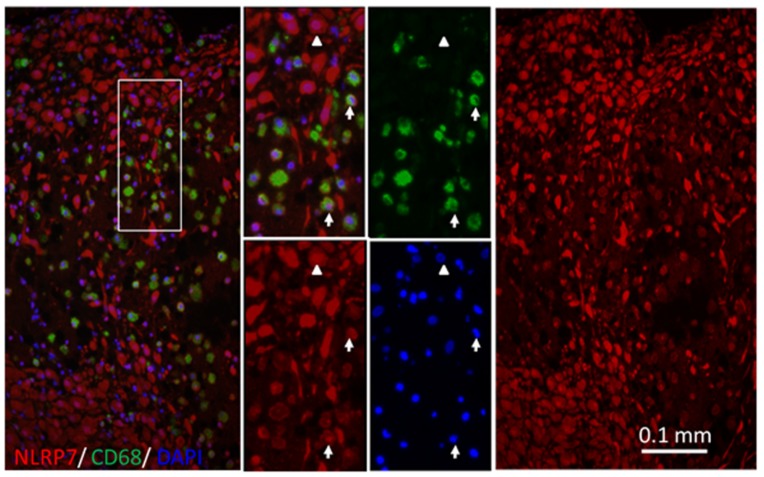
Colocalization of decidual macrophages in the human endometrium of the pregnant uterus. Immunofluorescent double staining of human endometrial tissue with anti-*NLRP7* antibodies (red), 4′,6-diamidino-2-phenylindole (DAPI) (blue), and antibodies against CD68 (green) for decidual macrophages. The outlined area is enlarged in the central panel. The arrows indicate CD68+/*NLRP7*+ (decidual macrophages) and the arrowheads indicate CD68−/*NLRP7*+ (Endometrial stromal cells). The colocalization of *NLRP7* was observed in CD68+ cells. Magnification ×200. Scale bar = 0.1 mm.

Our previous study found that *NLRP7* may contribute to the decidualization of endometrial stromal cells [18]. We went on to explore whether *NLRP7* plays a role in immune cells during pregnancy. In order to identify types of immune cells expressing *NLRP7*, we carried out immunofluorescent staining of CD3 (T cell marker) (Figure S1A), CD56 (NK cell marker) (Figure S1B), and CD68 (macrophage marker) (Figure 1 and Figure S1), respectively, combining with *NLRP7* on endometrial tissue of the first-trimester pregnancy. The *NLRP7* protein is up-regulated in the endometrial cells during pregnancy [18] and we found that *NLRP7* was abundant in endometrial tissues of the first-trimester pregnancy (Figure 1). Consistently with the previous report [18], we found that *NLRP7* was located in the nucleus and cytoplasm in the endometrial stromal cells (CD68− cells) (Figure 1). Interestingly, we found that *NLRP7* was co-localized with decidual macrophages (CD68+ cells) (Figure 1 and Figure S1). 

### 2.2. Macrophage Differentiation Increases NLRP7 Expression

**Figure 2:**
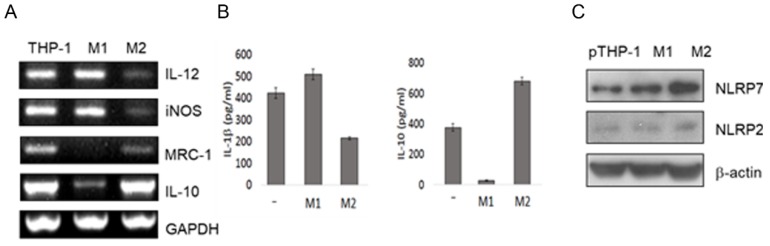
Protein expression in M1 and M2 macrophages. (**A**–**C**) The THP-1 cells were primed with 50 nM PMA for 6 h (denoted as pTHP-1). The pTHP-1 cells treated with 0.1 mg/mL LPS and 5 ng/mL IFN-γ for M1 differentiation (denoted as M1) or 25 ng/mL IL-4 and 25 ng/mL IL-13 for M2 differentiation (denoted as M2). The genes’ expressions were measured by semi-quantitative RT-PCR for M1-cell markers (IL-12 and iNOS) and M2-cell markers (MRC-1 and IL-10) (**A**). Supernatants were measured by ELISA for IL-1β and IL-10 production (**B**). The undifferentiated pTHP-1, M1 and M2 cells were harvested for the Western blot analysis of NLRP2, *NLRP7*, and β-actin protein levels (**C**).

M1 macrophages play a role in pro-inflammatory, whereas M2 macrophages play a role in anti-inflammatory during pregnancy [19]. In light of *NLRP7* expression in decidual macrophages, we next explored whether *NLRP7* is involved in macrophage differentiation. *NLRP7* has been identified in human primary macrophage [15] and THP-1 cells [20]. We attempted to differentiate M1 and M2 macrophages from THP-1, which is a human monocytic leukemia cell line from monocytic leukemia [21]. First, we confirmed the macrophage differentiation of the PMA primed THP-1 cells (designated as pTHP-1) under the standard induction factors. The results show that the IL-12 and insomnia had higher expression in pTHP-1 cells induced by LPS and IFN-γ (denoted as M1 macrophages), whereas the MRC-1, and IL-10 mRNA had a higher expression in pTHP-1 cells induced by IL-4 and IL-13 (denoted as M2 macrophages) (Figure 2A). The Enzyme-linked immunosorbent assay (ELISA) results confirm that M1 macrophages have higher IL-1β production, whereas M2 macrophages have higher IL-10 production (Figure 2B). Taken together, these findings confirm that the pTHP-1 cells differentiate to M1 and M2 lineages (Figure 2A,B). Next, we examined *NLRP7* expression in the M1 and M2 lineages. A Western blot analysis showed that the *NLRP7* protein level was higher in M1 and M2 macrophages than in the undifferentiated pTHP-1 cells (denoted as pTHP-1) (Figure 2C). In addition, a higher level of *NLRP7* was found in the M2 macrophages than in M1 macrophages (Figure 2C). In contrast, NLRP2 protein level showed no apparent differences between pTHP-1, M1, and M2 macrophages (Figure 2C). Together, our results suggest that *NLRP7* is associated with M2 macrophage differentiation.

### 2.3. Ectopic Expression of NLRP7 in Macrophage Results in M2 Polarization

**Figure 3:**
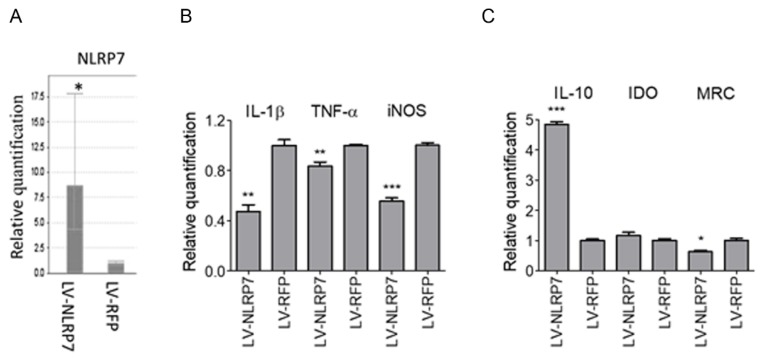
Expression of *NLRP7* in macrophage results in M2 polarization. (**A**) THP-1 cells were infected with LV-*NLRP7* or LV-RFP. The *NLRP7* expression was confirmed by qPCR. (**B**,**C**) The infected cells were primed with PMA followed by treatment with 0.1 mg/mL LPS and 5 ng/mL IFN-γ for M1 differentiation (**B**) or treatment with 25 ng/mL IL-4 and 25 ng/mL IL-13 for M2 differentiation (**C**). The cells were collected for detecting the expressions of M1-markers (IL-1β, TNF-α, and iNOS) (**B**) and M2-markers (IL-10, IDO, and MRC) (**C**). IL-1β and IL-10 were measured by ELISA, while TNF-α, iNOS, IDO, and MRC were measured by qRT-PCR. * *p* < 0.05, ** *p* < 0.005, and *** *p* < 0.0005, Student’s t-test. Data are representative of at least two or three independent experiments.

In light of the evidence, genetic variants of *NLRP7* were associated with recurrent miscarriage [22]. Consequently, we speculate that *NLRP7* plays a protective role in the maintenance of normal pregnancy. During pregnancy, M2 macrophages are important to sustain fetomaternal tolerance [19]. Therefore, we investigated whether *NLRP7* is involved in M2 polarization. THP-1 cells and pTHP-1 cells over-expressing *NLRP7* were induced toward the M1 or M2 lineages. The qRT-PCR of *NLRP7* transcripts (Figure 3A) showed that the NLRP-lentivirus infected THP-1 cells (denoted as LV-*NLRP7*) had eight folds of *NLRP7* mRNA expression over THP-1 cells over the mock control (denoted as LV-RFP). This result confirms the over-expression of in transected cells. Furthermore, we examined the expressions of M1 macrophage markers (IL-1β, TNF-α, and iNOS) and M2 macrophage markers (IL-10, IDO, and MRC), respectively, in these cells. We found lower levels of IL-1β, TNF-α, and iNOS (M1 macrophage markers) in LV-*NLRP7* infected pTHP-1 cells than in the mock control (Figure 3B). IL10, an M2 macrophage marker, was higher in LV-*NLRP7* infected pTHP-1 cells than the mock control (Figure 3C). But other M2 macrophage markers, (IDO and MRC) did not present a significant difference. These findings suggest that *NLRP7* over-expression suppresses the expression of M1 markers (IL-1β, TNF-α, and iNOS), enhances the expression of the M2 marker (IL-10), and might interfere with M1/M2 differentiation.

### 2.4. NLRP7 Are More Prevalent in the Decidual M2 Macrophages in the Human Endometrium

**Figure 4:**
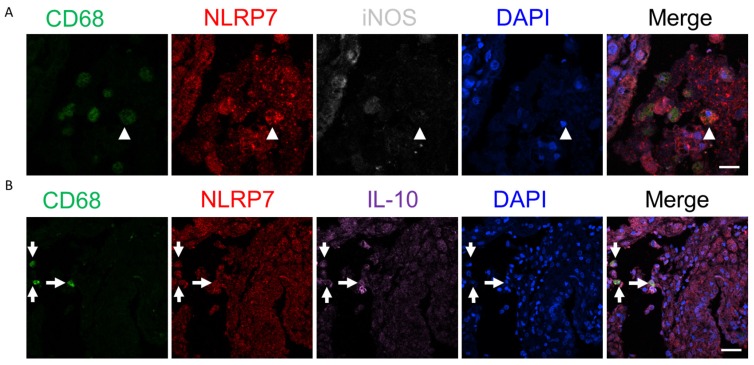
*NLRP7* is predominantly expressed in decidual M2 macrophages (CD68+/IL-10+) in the human endometrium of the pregnant uterus. (**A**,**B**) Immunofluorescent triple staining of endometrial tissue with anti-*NLRP7* antibodies (red, OPAL-570), 4′,6-diamidino-2-phenylindole (DAPI) (blue), antibodies against CD68 for decidual macrophages (green, OPAL-520), and antibodies against iNOS for M1 macrophages (white, OPAL-690) (**A**) and antibodies against IL-10 for M2 macrophages (purple, OPAL-690) (**B**). The arrowheads indicate CD68+/*NLRP7*+/iNOS+ (decidual M1 macrophages), and the arrows indicate CD68+/*NLRP7*+/IL-10+ cells (decidual M2 macrophages). Magnification ×200. Scale bar = 20 μm.

**Figure 5:**
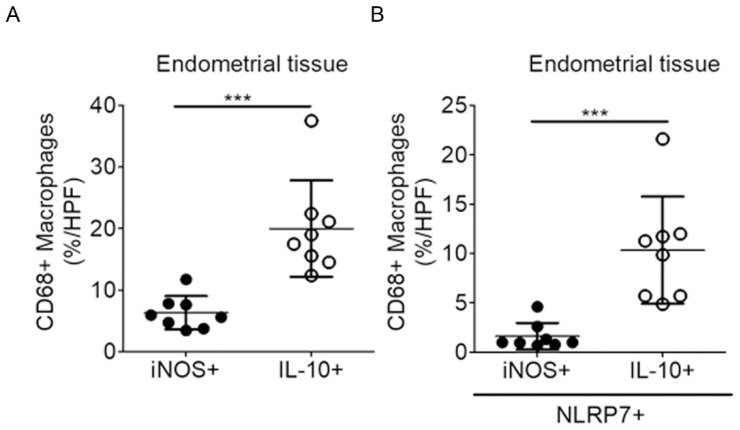
M2 macrophages are more prevalent than the *NLRP7*+ M1 macrophages in the human endometrium of the pregnant uterus. (**A**,**B**) Endometrial tissues were stained by the OPAL kit for *NLRP7*, CD68, iNOS, IL-10, and DAPI. Data presented as dot plots show the percentage of M1 (CD68+/iNOS+) or M2 (CD68+/IL-10+) macrophages within a high-powered field (HPF) (**A**). Data presented as dot plots show the percentage of *NLRP7*+ M1 or *NLRP7*+ M2 macrophages within a high-powered field (HPF) (**B**). In all of the quantitative experiments, *n* = 8 pregnant women and at least 10 high-power fields. Data are mean ± SD. *** *p* < 0.0005 compared to M1 macrophages.

Having shown the involvement of *NLRP7* in the macrophage differentiation, we next explored the expression pattern of *NLRP7* in the first-trimester decidual macrophage. Endometrial tissues from eight pregnant women were used to analyze the number and proportion of *NLRP7*+ decidual macrophages. In the beginning, we carried out double immunofluorescent staining of endometrial tissue using CD86 (an M1 marker) and CD206 (an M2 marker), combining with *NLRP7* in the endometrial samples. We observed the red signals of *NLRP7* co-localized with the green signals of CD86 (Figure S2A) or CD206 (Figure S2B). For accurate quantification of NLRP positive cells in the M1 and M2 decidual macrophages, respectively, we carried out triple immunofluorescent staining of endometrial tissue using a combination of CD68 (macrophage marker), *NLRP7*, iNOS (M1 marker), or IL-10 (M2 marker). DAPI (4′,6-diamidino-2-phenylindole, blue) was used to label the nuclei. The CD68 positive cells (green, OPAL-520) expressing *NLRP7* (red, OPAL-570) and iNOS (white, OPAL-690) indicated the decidual M1 macrophages (Figure 4A). The CD68 positive cells expressing *NLRP7* and IL-10 (purple, OPAL-690) indicated decidual M2 macrophages (Figure 4B). IL-10 is an anti-inflammatory cytokine and plays an important role in a successful pregnancy. During pregnancy, IL-10 is synthesized in both immune and non-immune cells [23,24]. Indeed, we detected abundant IL-10 signals in the endometrial tissue. We found that the M2 macrophages were indeed more prevalent than the M1 macrophages (Figure 5A). In addition, the *NLRP7* positive M2 macrophages (IL-10+/CD68+/*NLRP7*+ cells) were more prevalent than the *NLRP7* positive M1 macrophages (iNOS+/CD68+/*NLRP7*+ cells) (Figure 5B). Taken together, these findings suggest that *NLRP7* is predominately expressed in the decidual M2 macrophages, as compared with the M1 macrophages.

## 3. Discussion

*NLRP7* belongs to the NLRP family and is located on human chromosome 19q13.4. It plays multiple roles including immunity and reproduction [25]. *NLRP7* is present only in primates. It probably emerged from gene duplication of NLRP2 during evolution [26]. Therefore, *NLRP7* has been referred to as a primate-specific NLRP gene.

Controversies exist regarding the role of *NLRP7* in immunity and inflammation. Given that the overexpression of *NLRP7* in HEK-293T cells impaired caspase-1-mediated IL-1β production, *NLRP7* was described as an inhibitor of the inflammasome signaling pathway [20]. However, subsequent studies showed that *NLRP7* induced an inflammasome formation as well as inflammatory cytokines production in response to microbial antigens [15,16,27]. In peripheral blood mononuclear cells, *NLRP7* was associated with the Golgi apparatus and microtubules, suggesting that it may coordinate cytokines transport and secretion [17]. The contradiction may reflect the plasticity of *NLRP7*, which prevents inflammasome formation and IL-1β release in certain cell types while activating the inflammasome in response to infections in other cell types [28].

In addition to the “canonical” role in inflammation and immunity, *NLRP7* has important functions related to genesis and embryogenesis, which may be independent of inflammasome signaling. *NLRP7* transcripts have been detected in the endometrium, placenta, hematopoietic cells, oocytes and preimplantation embryos [29]. *NLRP7* is usually referred to as a maternal effect gene, whose mutations result in miscarriage, stillbirth, fetal growth restriction, preeclampsia, and imprinting disorders [17,29,30,31,32,33,34]. *NLRP7* and KHDC3L, a component of subcortical maternal complex (SCMC), have been co-localized to the oocyte cytoskeleton and became predominant at the cortical region in growing oocytes [35]. SCMC is a multiprotein complex uniquely expressed in mammalian oocytes and is essential for zygotes to progress beyond the first embryonic cell divisions. *NLRP7* has also been proposed to function in chromatin reprogramming and DNA methylation in the germline or early embryonic development [36]. Given that *NLRP7* expresses during ovarian follicle development and early embryo development, *NLRP7* may have an essential role in oogenesis and embryogenesis as well.

Previous studies reported that *NLRP7* was expressed in human immune cell lines of B, T, and monocytic cells [30], as well as in a variety of human tissues, including the lung, spleen, thymus, and reproductive organs (testis and ovaries) [20,26]. For the first time, we found that *NLRP7* was expressed in the decidual macrophage during the first-trimester pregnancy. We also found that *NLRP7* positive cells are more prevalent in the decidual M2 macrophages. Using an in vitro differentiation model, we found that *NLRP7* was up-regulated in both M1 and M2 lineages after induction, but the up-regulation is more pronounced in the M2 lineage. In addition, the over-expression of *NLRP7* suppressed the production of the M1 macrophage cytokines and enhanced the production of the M2 macrophage cytokines. Although *NLRP7* seems to be involved in the decidual macrophage differentiation, the precise mechanism remains obscure. Our finding seems to be in accordance with the observation of normal *NLRP7* inhibited pro-IL1β synthesis in peripheral blood mononuclear cells [17,30].

During human pregnancy, macrophages comprise approximately 20% of the leukocyte population at the implantation sites. The macrophages polarize into M1 macrophages in the peri-implantation period. The decidual macrophages shift toward a predominantly M2 phenotype after the placental development is completed. The M2 macrophages promote maternal immune tolerance to semiallogeneic fetuses until parturition [12]. Studies have shown that M-CSF, IL-10, IL33, IL34, CXCL16, HLA-G5, and VEGF are able to polarize monocyte to the M2-like decidual macrophages [37,38,39,40,41,42,43,44]. In this study, we showed that *NLRP7* is involved in the differentiation of decidual macrophages, with prominent *NLRP7* expression in the M2 lineage. How *NLRP7* regulates differentiation of decidual macrophages requires further investigation. Macrophages are important in promoting and maintaining homeostasis at the fetal-maternal interface [45]. Considering that *NLRP7* relates to the decidualization [18] and macrophage differentiation, we propose that *NLRP7* is a primate-specific multitasking gene to maintain endometrial homeostasis and reproductive success.

## 4. Materials and Methods

### 4.1. Tissue Samples and Cells

This study was approved by the Institutional Review Board of National Cheng Kung University Hospital (NCKUH, No.: A-ER-102-440; 03 March 2014). Endometrial tissues were acquired from pregnant women who had undergone elective termination of pregnancy for non-medical reasons (*n* = eight; ages ranging from 25–36; gestational ages at sampling ranging from 8–12 weeks). Samples were obtained by dilatation and evacuation without any prior pharmaceutical induction. In all the samples, chromosomal abnormalities of the products of conception (POC) were excluded. Cases with possible intra-uterine infection were also excluded. Following the evacuation, specimens were immediately immersed in formalin and embedded in a snap and sectioned with a cryostat (Leica, Wetzlar, Germany). The human monocytic cell line THP-1 obtained from the American Type Culture Collection (ATCC, Manassas, VA, USA) was cultured in RPMI 1640 medium (Thermo Fisher Scientific, Waltham, MA, USA) supplemented with 10% fetal bovine serum (FBS) and 1% antibiotic-antimycotic solution (100 units/mL of penicillin, 100 mg/mL of streptomycin, and 0.25 mg/mL of Fungizone) (Life technologies). THP-1 was grown in an incubator at 37 °C under a 5% CO_2_ atmosphere at constant humidity.

### 4.2. Reagents

Rabbit anti-human *NLRP7* antibody (IMG-6357A) was obtained from Novus Biologicals (Littleton, CO, USA). Mouse anti-human NLRP2 (sc-166584), mouse anti-humanβ-actin (sc-47778), mouse anti-CD206 (sc-376108) antibodies were procured from Santa Cruz Biotechnology (Santa Cruz, CA, USA). Mouse anti-human CD68 (NCL-CD68-KP1), mouse anti-human CD3 (NCL-L-CD3-565), and mouse anti-human CD56 (NCL-L-CD56-1B6) antibodies were procured from Leica Microsystems (Newcastle upon Tyne, UK). Rabbit anti-iNOS (bs-2072R) and IL-10 (bs-0698R) were obtained from Bioss (Beijing, China). Mouse anti-CD86 (GTX74650) was acquired from GeneTex (Irvine, CA, USA). Alexa 488-conjugated goat anti-mouse IgG (A28175), Alexa 488-conjugated goat anti-rabbit IgG(A11008), and Alexa 568-conjugated goat anti-rabbit IgG (A-11011) were obtained from Thermo Fisher Scientific (Waltham, MA, USA). Goat Anti-Mouse IgG H&L (Alexa Fluor^®^ 568) was obtained from Abcam (Cambridge, MA, USA). Mounting medium DAPI Fluoromount-G (0100–20) for immunofluorescence was obtained from SouthernBiotech (Birmingham, AL, USA). ELISA kits of human IL-1β (88-7261) and IL-10 (88-7106) were purchased from eBioscience. IFN-γ (GFH77), IL4 (GFH9), and IL13 (GFH85) were purchased from Cell Guidance Systems (St. Louis, MO, USA). LPS (tlrl-peklps) was purchased from Invivogen (San Diego, CA, USA). Phorbol 12-myristate 13-acetate (PMA) (P8139) and citrate buffer (C9999, Sigma) were purchased from Sigma (St. Louis, MO, USA). Peroxidase conjugated goat anti-mouse IgG was obtained from Jackson Immuno-Research Laboratories (West Grove, PA, USA), while ECL plus reagent was purchased from Amersham Biosciences (Piscataway, NJ, USA). A Direct-zol RNA MiniPrep kit (R2050) was acquired from Zymo Research (Irvine, CA, USA).

### 4.3. Lentivirus Preparation and Infection

pLAS2w.Pneo-*NLRP7* and pLAS2w.RFP-C.Pneo plasmids were described previously [18]. *NLRP7*-expressed lentivirus (LV-*NLRP7*) and control RFP-expressed lentivirus (LV-RFP) were prepared by RNAi Core Facility (National Cheng Kung University Hospital, Tainan, Taiwan). Then, THP-1 cells were infected with LV-*NLRP7* or LV-RFP overnight and then selected by puromycin (0.5 μg/mL) for two weeks.

### 4.4. M1 and M2 Macrophage Differentiation of THP-1 Cells

The THP-1 cells were primed with 50 nM PMA for 6 h. For M1 macrophage differentiation, the cells were treated with 0.1 mg/mL LPS and 5 ng/mL IFN-γ. For M2 macrophage differentiation, the cells treated with 25 ng/mL IL-4 and 25 ng/mL IL-13.

### 4.5. ELISA

The supernatants of the differentiated macrophages as described above were collected for detecting the IL-1βand IL-10 by ELISA.

### 4.6. RT-PCR and Quantitative Real-Time PCR

RNA samples of the differentiated macrophages were isolated by the Direct-zol RNA MiniPrep kit, with 2 μg of each sample being subjected to reverse transcription into cDNA. 

For RT-PCR, cDNA was amplified by PCR using specific primers (human IL-12 sense 5′-TGATGACATCAAGAAGGTGGTGAAG-3′; human IL-12 antisense 5′-TCCTTGGAGGCCATGTGGGCCAT-3′; human iNOS sense 5′-CTCCTCCAAATTGCTCTCCT-3′; human iNOS antisense 5′-CCTTGGCCTTCAGGTAATGC-3′; human MRC-1 sense 5′-CTCCTCCAAATTGCTCTCCT-3′; human MRC-1 antisense 5′-CCTTGGCCTTCAGGTAATGC-3′; human IL-10 sense 5′-CTCCTCCAAATTGCTCTCCT-3′; human IL-10antisense 5′-CCTTGGCCTTCAGGTAATGC-3′ human GAPDH sense 5′-GGCGTCTTCACCACCAT-3′; human GAPDH antisense 5′-CACCACCCTGTTGCTGTA-3′).

The quantitative PCR was performed using Fast SYBR Green Master Mix (Thermo Fisher Scientific, city, LA, USA) following the manufacturer’s instructions. The quantitative PCR analysis was performed with the StepOne Plus real-time PCR apparatus (Thermo Fisher Scientific). Primers used for this study included: *NLRP7* forward primer: 5′-CTTCTGTGCGGATTCTTTGTGA-3′; *NLRP7* reverse primer: 5′-TTTTTAATCTCCACTTTCTGCAGATG-3′; iNOS forward primer: 5′-CCCCCAGCCTCAAGTCTTATT-3′; iNOS reverse primer: 5′-GAGAGGAGGCTCCGATCAATC-3′; IDO forward primer: 5′-TTTCACCAAATCCACGATCA-3′; IDO reverse primer: 5′-TGCAAACTCCTTTTGGGTCTT-3′; TNF-α forward primer: 5′-GGCGGTGCTTGTTCCTCAG-3′; TNF-α reverse primer: 5′-CTCTCAGCTCCACGCCATTG-3′; MRC1 forward primer: 5′-GGCGGTGACCTCACAAGTATCC-3′; MRC1 reverse primer: 5′-CCGATCTGCCCAGTACCCATC-3′; β-actin forward primer: 5′-CATGTACGTTGCTATCCAGGC-3′; β-actin reverse primer: 5′-CTCCTTAATGTCACGCACGAT-3′. The data presented were normalized to β-actin. 

### 4.7. Western Blotting

The THP-1 cells were collected for extracting cell lysate. The cell lysates were combined with 6X protein loading buffer and denatured in boiled water for 10 min. The treated cell lysates were then subjected to SDS-PAGE and thereafter transferred to PVDF membranes. After blocking, the membranes were incubated with the primary antibodies NLRP2, *NLRP7*, and β–actin at 4 °C overnight. Bands were visualized using peroxidase-conjugated goat anti-mouse IgG and ECL plus reagents.

### 4.8. Immunofluorescence Staining

Tissue sections (5-μm-thick) were prepared from the endometrium of women who underwent elective termination of pregnancy. The 5 μm tissue sections of the paraffin-embedded tissue underwent deparaffinization and rehydration. Slides were subjected to heat-induced antigen retrieval (citrate buffer pH 6.0 for 20 min).

Slides were incubated with a blocking solution containing 3% BSA in Dako antibody diluent (S302283, DAKO, Denmark) for 30 min at room temperature and then incubated with primary antibodies overnight in a humidified chamber at 4 °C. The following day, slides were incubated with Alexa 488-conjugated secondary antibody and Alexa 568-conjugated secondary antibody for 2 h in a humidified chamber at room temperature and mounted in DAPI Fluoromount-G.

Triple immunofluorescence staining was performed using the Opal 4-Color Manual IHC Kit (Cat#NEL810001KT; PerkinElmer, Waltham, MA, USA), according to the manufacturer’s instructions. After deparaffinization, slides were placed in antigen retrieval (AR) buffer and boiled using a microwave oven (Whirlpool, Taipei, Taiwan). Endogenous peroxidase activity was blocked by hydrogen peroxidase. Following blocking to eliminate nonspecific binding, slides were incubated with a primary antibody overnight in a humidified chamber at 4 °C. The following day, the slides were then washed and incubated with Opal Polymer HRP Ms + Rb (Cat#ARH1001EA; PerkinElmer, Waltham, MA, USA). Then, the slides were incubated with one of the following fluorescent TSA^®^ reagents included in the kit (PerkinElmer, Waltham, MA, USA) to detect each antibody staining: Opal 520, Opal 570, or Opal 690. For labeling of the next primary antibody, each previous primary and secondary antibody was removed by boiling in AR buffer. Slides were incubated with the next primary and Opal Polymer HRP Ms + Rb and slides were subsequently incubated with the next fluorescent TSA^®^ reagents. These steps were repeated until all three markers were labeled, then the slides were mounted in DAPI Fluoromount-G. To quantify the fluorescent signals, the percentage of M1 and M2 in at least 10 fields under 200× magnification for each case.

### 4.9. Statistics

The results of ELISA and qRT-PCR are represented as means ± SD. Differences between groups were assessed by the student’s *t*-test. A *p*-value of less than 0.05 is considered statistically significant.

## Reference

## Supplementary Materials

The following are available online at https://www.mdpi.com/1422-0067/20/23/5994/s1.
