# Supplementary material for: NLRP7 Is Involved in the Differentiation of the Decidual Macrophages
Source: Int J Mol Sci. 2019 Nov 28;20(23):5994. doi: 10.3390/ijms20235994 (PMC6929161; doi:10.3390/ijms20235994)
Supplement: Supplementary file 1 [file ijms-20-05994-s001.pdf]

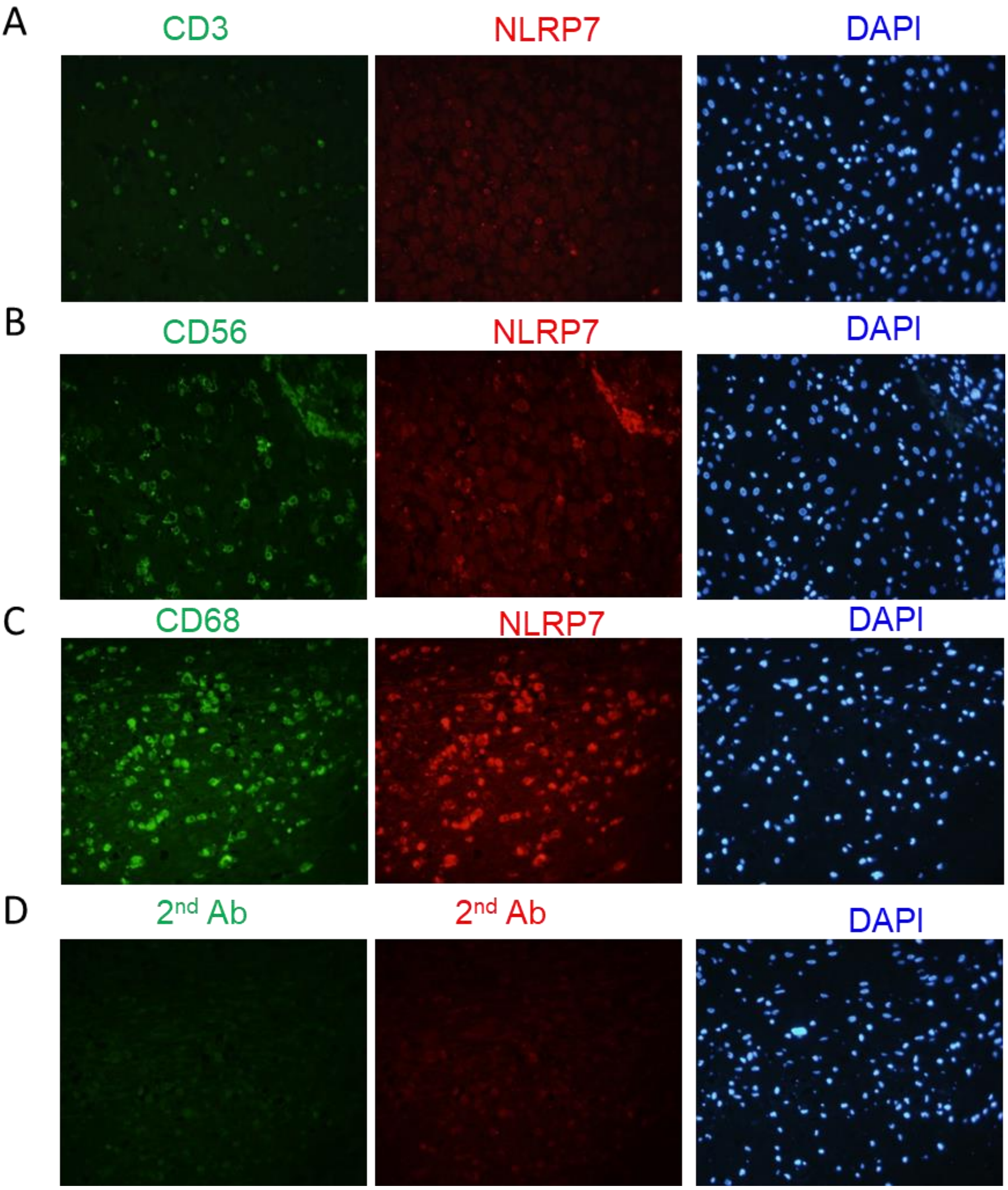

Figure S1. NLRP7 is predominantly expressed in CD68+ decidual macrophages in the human endometrium of the pregnant uterus. (A-D) Immunofluorescent double staining of endometrial tissue with anti-NLRP7 antibodies (red), 4',6-diamidino-2-phenylindole (DAPI) (blue) and antibodies against cellular marker (green) for T lymphocytes, CD3 (A); NK cells, CD56 (B); and decidual macrophages, CD68 (C). The 2nd antibody staining served as control (D). Magnification ×200.

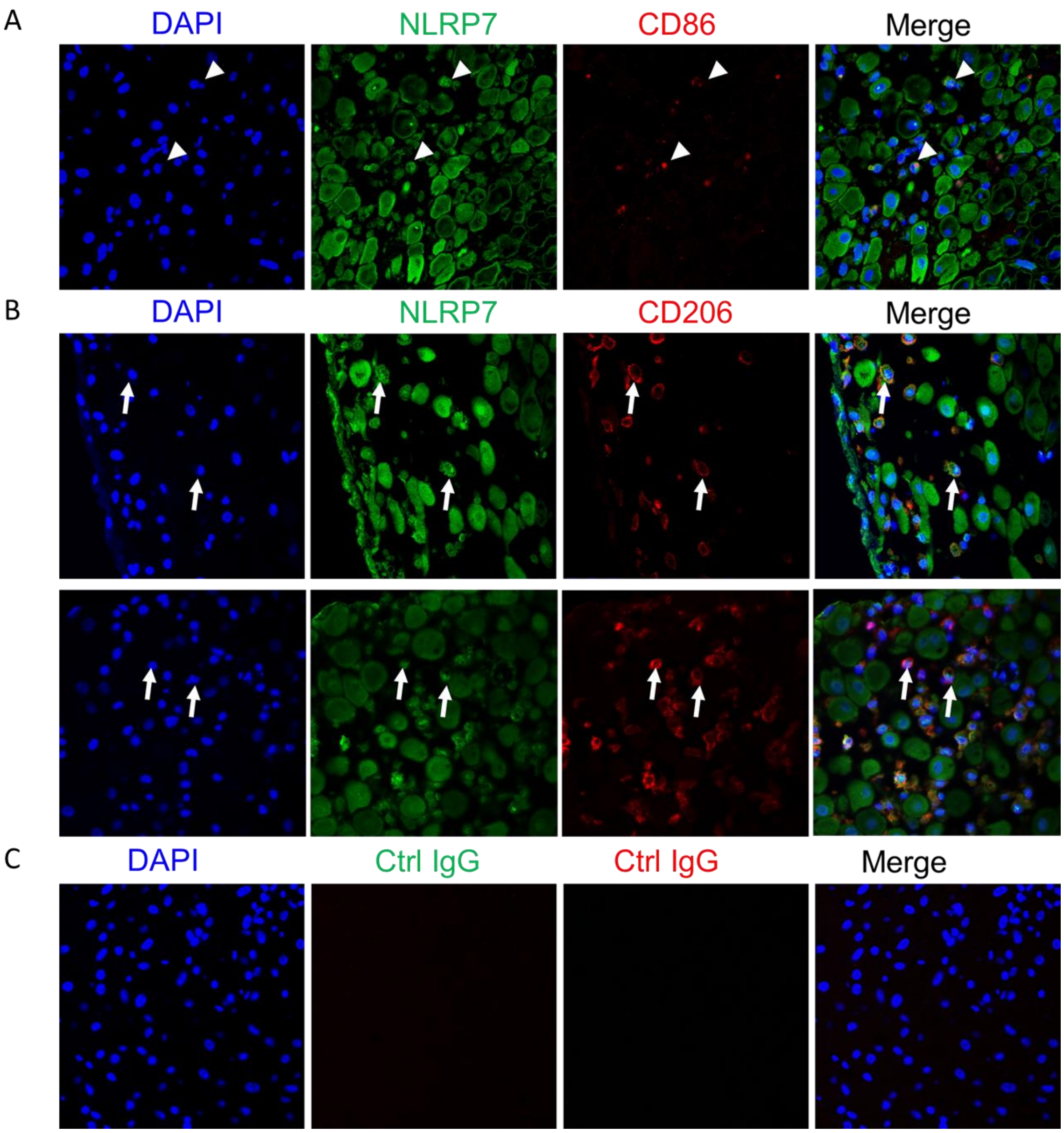

Figure S2. NLRP7 is predominantly expressed in M2 decidual macrophages in the human endometrium of the pregnant uterus. (A-C) Immunofluorescent double staining of endometrial tissue with anti-NLRP7 antibodies (green), 4′,6-diamidino-2-phenylindole (DAPI) (blue) and antibodies against cellular marker (red) for M1 macrophages, CD86 (A); and M2 macrophages, CD206 (B). The control (Ctrl) IgG antibody staining served as control (D). Arrowheads indicate CD86+/NLRP7+ (decidual M1 macrophages), and arrows indicate CD206+/NLRP7+ cells (decidual M2 macrophages). The colocalization of NLRP7 was observed in CD206+ cells. Magnification ×600.
